# Copper(II) Bis(diethyldithiocarbamate) Induces the Expression of Syndecan-4, a Transmembrane Heparan Sulfate Proteoglycan, via p38 MAPK Activation in Vascular Endothelial Cells

**DOI:** 10.3390/ijms19113302

**Published:** 2018-10-24

**Authors:** Takato Hara, Hiroko Tatsuishi, Tomomi Banno, Tomoya Fujie, Chika Yamamoto, Hiroshi Naka, Toshiyuki Kaji

**Affiliations:** 1 Department of Environmental Health, Faculty of Pharmaceutical Sciences, Tokyo University of Science, 2641 Yamazaki, Noda 278-8510, Japan; takato.hara@phar.toho-u.ac.jp (T.H.); j3a12066@ed.tus.ac.jp (H.T.); 2 Department of Environmental Health, Faculty of Pharmaceutical Sciences, Toho University, 2-2-1 Miyama, Funabashi 274-8510, Japan; t-fujie@phar.toho-u.ac.jp (T.F.); yamamoto@phar.toho-u.ac.jp (C.Y.); 3 Graduate School of Science, Nagoya University, Furo-cho, Chikusa-ku, Nagoya 464-8602, Japan; banno.tomomi@a.mbox.nagoya-u.ac.jp; 4 Research Center for Materials Science, Nagoya University, Furo-cho, Chikusa-ku, Nagoya 464-8602, Japan; h_naka@nagoya-u.jp

**Keywords:** bioorganometallics, metal coordination complexes, organocopper compound, proteoglycan, syndecan-4, vascular endothelial cell

## Abstract

Proteoglycans synthesized by vascular endothelial cells are important for regulating cell function and the blood coagulation-fibrinolytic system. Since we recently reported that copper(II) bis(diethyldithiocarbamate) (Cu(edtc)_2_) modulates the expression of some molecules involving the antioxidant and blood coagulation systems, we hypothesized that Cu(edtc)_2_ may regulate the expression of proteoglycans and examined this hypothesis using a bovine aortic endothelial cell culture system. The experiments showed that Cu(edtc)_2_ induced the expression of syndecan-4, a transmembrane heparan sulfate proteoglycan, in a dose- and time-dependent manner. This induction required the whole structure of Cu(edtc)_2_—the specific combination of intramolecular copper and a diethyldithiocarbamate structure—as the ligand. Additionally, the syndecan-4 induction by Cu(edtc)_2_ depended on the activation of p38 mitogen-activated protein kinase (MAPK) but not the Smad2/3, NF-E2-related factor2 (Nrf2), or epidermal growth factor receptor (EGFR) pathways. p38 MAPK may be a key molecule for inducing the expression of syndecan-4 in vascular endothelial cells.

## 1. Introduction

Proteoglycans are extracellular macromolecules that consist of a core protein and one or more glycosaminoglycan side chains [1]. Proteoglycans serve as signal mediators by binding to biological factors such as growth factors, cytokines, and lipids via the core protein or glycosaminoglycan side chains to modulate the biological activities of these factors [2,3]. The vascular organs are rich in proteoglycans, and the proteoglycans synthesized from vascular endothelial cells that line the inner surface of blood vessels have physiological functions such as anticoagulant activity [4,5], permeability [6], lipid metabolism [7], and extracellular matrix assembly [8].

Cells synthesize two major types of proteoglycans: heparan sulfate proteoglycans and chondroitin/dermatan sulfate proteoglycans [9]. The former includes a large extracellular matrix proteoglycan, perlecan, which exists in the basement membrane [10], and the transmembrane heparan sulfate proteoglycan syndecan family [11]. The latter includes the small leucine-rich proteoglycan biglycan [12]. Syndecan-4 was found in rat foot pad endothelial cells as a proteoglycan that binds antithrombin III via its heparan sulfate chains [11,13]. The transmembrane heparan sulfate proteoglycan is importantly involved in the focal adhesion and alignment of vascular endothelial cells along the direction of blood flow [14]. In addition, since syndecan-4 is also involved in wound healing and cellular defense mechanisms [15,16], clarifying the regulation of syndecan-4 expression is important for understanding the molecular mechanisms underlying the physiological function of endothelial cells.

Metal coordination complexes consist of metal(s) and organic structure(s) as the common features. For a long time, the applications of metal coordination complexes were limited in studies on chemical reaction; however, it has been found that certain hybrid molecules can exhibit unique biological activities that cannot be induced by either their intramolecular metal or organic structure alone [17]. Recently, we reported that copper(II) bis(diethyldithiocarbamate) (termed Cu(edtc)_2_) is an inducer of NF-E2-related factor2 (Nrf2) [18] and analyzed the function of vascular endothelial cells using Cu(edtc)_2_ [19,20]. In the present study, we investigated whether Cu(edtc)_2_ modulates the expression of syndecan-4 and other proteoglycans in vascular endothelial cells. It was revealed that Cu(edtc)_2_ induces endothelial syndecan-4 expression via the activation of p38 mitogen-activated protein kinase (MAPK).

## 2. Results

### 2.1. Cu(edtc)_2_ Induces Syndecan-4 Expression in Vascular Endothelial Cells

**Figure 1:**
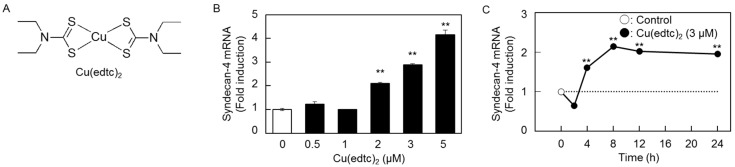
Induction of *syndecan-4* mRNA by Cu(edtc)_2_ treatment in vascular endothelial cells. (**A**) The structure of Cu(edtc)_2_. The expression of *syndecan-4* mRNA; the cells were incubated at 37 °C (**B**) for 8 h in the presence of Cu(edtc)_2_ (0.5, 1, 2, 3, and 5 µM) or (**C**) for 2, 4, 8, 12, and 24 h in the presence of Cu(edtc)_2_ (3 µM). *Syndecan-4* mRNA levels were then determined by real-time reverse transcription polymerase chain reaction (RT-PCR). Values are means ± S.E. of four replicates. ** *p* < 0.01 vs. the corresponding control.

Figure 1 shows the expression of *syndecan-4* mRNA in vascular endothelial cells treated with Cu(edtc)_2_ (Figure 1A). *Syndecan-4* mRNA was upregulated by Cu(edtc)_2_ at concentrations at and above 2 µM in a dose-dependent manner after an 8-h treatment (Figure 1B). Cu(edtc)_2_ at 3 µM significantly increased the expression of *syndecan-4* mRNA after 4 h or longer in a time-dependent manner (Figure 1C).

**Figure 2:**
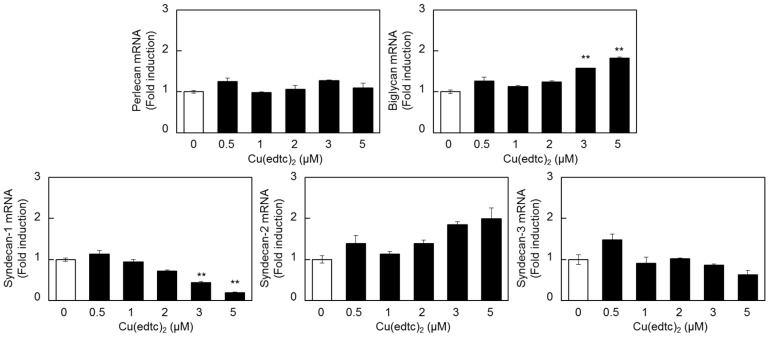
Effects of Cu(edtc)_2_ on the expression of *perlecan*, *biglycan*, *syndecan-1*, *syndecan-2*, and *syndecan-3* mRNA in vascular endothelial cells. The cells were incubated at 37 °C for 8 h in the presence of Cu(edtc)_2_ (0.5, 1, 2, 3, and 5 µM). Proteoglycan mRNA levels were then determined by RT-PCR. Values are means ± S.E. of four replicates. ** *p* < 0.01 vs. control.

The expression of mRNAs other than that for *syndecan-4* in vascular endothelial cells treated with Cu(edtc)_2_ was investigated (Figure 2). Cu(edtc)_2_ upregulated the expression of *biglycan* and *syndecan-2* mRNAs; however, they were not upregulated as strongly as *syndecan-4*. On the other hand, only *syndecan-1* mRNA was reduced by Cu(edtc)_2_ treatment at 8 h in a dose-dependent manner.

### 2.2. Characterization of Upregulation of Syndecan-4 mRNA Expression by Cu(edtc)_2_

**Figure 3:**
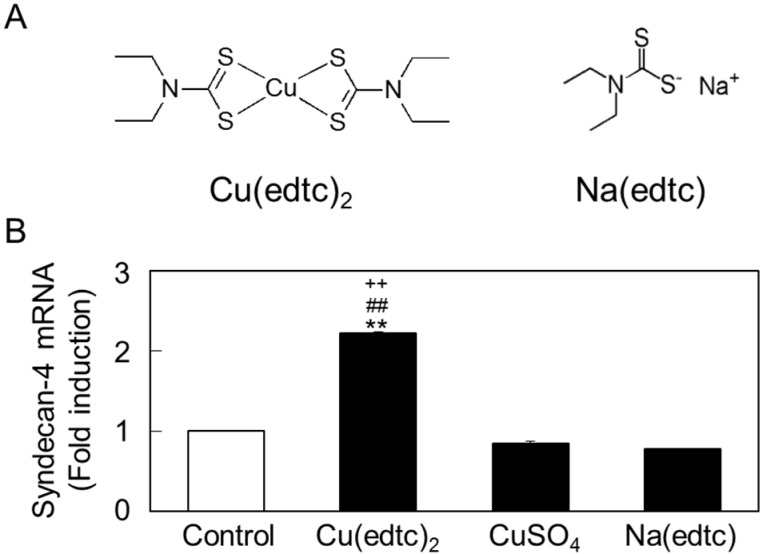
Comparison of the *syndecan-4* mRNA induction by different structural components of Cu(edtc)_2_. (**A**) Structure of Cu(edtc)_2_ and Na(edtc); (**B**) expression of *syndecan-4* mRNA. The cells were incubated at 37 °C for 8 h in the presence of Cu(edtc)_2_ (3 µM), CuSO_4_ (3 µM), or Na(edtc) (6 µM). *Syndecan-4* mRNA levels were then determined by RT-PCR. Values are means ± S.E. of four replicates. ** *p* < 0.01 vs. the corresponding control. ^##^ *p* < 0.01 vs. CuSO4. ^++^ *p* < 0.01 vs. Na(edtc).

To investigate how the functional structure of Cu(edtc)_2_ induces the expression of *syndecan-4* mRNA, vascular endothelial cells were treated with Cu(edtc)_2_, CuSO_4_, or sodium diethyldithiocarbamate (Na(edtc)), the ligand of Cu(edtc)_2_ (Figure 3A). As shown in Figure 3B, unlike Cu(edtc)_2_, both inorganic copper and the ligand failed to upregulate *syndecan-4* mRNA expression. This result indicated that the whole structure of Cu(edtc)_2_ as a metal coordination complex is required to induce the expression of *syndecan-4* mRNA.

**Figure 4:**
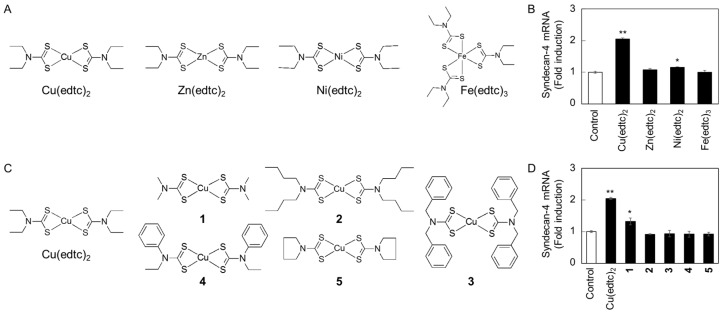
Role of copper and EDTC in the Cu(edtc)_2_ molecule in *syndecan-4* mRNA induction in vascular endothelial cells. (**A**) Structure of Cu(edtc)_2_, Zn(edtc)_2_, Ni(edtc)_2_, and Fe(edtc)_3_; (**B**) expression of *syndecan-4* mRNA. The cells were incubated at 37 °C for 8 h in the presence of Cu(edtc)_2_, Zn(edtc)_2_, Ni(edtc)_2_, or Fe(edtc)_3_ (3 µM each), then *syndecan-4* mRNA levels were measured by RT-PCR; (**C**) structure of Cu(edtc)_2_, and copper complexes **1**–**5**; (**D**) expression of *syndecan-4* mRNA. The cells were incubated at 37 °C for 8 h in the presence of copper complexes (3 µM each), then *syndecan-4* mRNA levels were measured by RT-PCR. Values are means ± S.E. of four replicates. * *p* < 0.05; ** *p* < 0.01 vs. control.

To examine whether either intramolecular copper or EDTC is essential for inducing endothelial *syndecan-4* expression, the effects of zinc, nickel, and iron complexes with EDTC (Zn(edtc)_2_, Ni(edtc)_2_, and Fe(edtc)_3_, respectively) on the *syndecan-4* mRNA expression were investigated (Figure 4). Neither Zn(edtc)_2_, Ni(edtc)_2_, nor Fe(edtc)_3_ significantly increased the expression of *syndecan-4* mRNA in vascular endothelial cells, although the expression was induced by Cu(edtc)_2_ (Figure 4A,B), suggesting that intramolecular copper is involved in inducing endothelial *syndecan-4* mRNA expression. On the other hand, copper complexes with ligands different from that of Cu(edtc)_2_ (copper(II) bis(dibutyldithiocarbamate) (**2**), copper(II) bis(dibenzyldithiocarbamate) (**3**), copper(II) bis(*N*-ethyl-*N*-phenyldithiocarbamate) (**4**), and copper(II) bis(pyrrolidinedithiocarbamate) (**5**)) failed to increase the expression of *syndecan-4* mRNA in vascular endothelial cells, while copper(II) bis(dimethyldithiocarbamate) (**1**) slightly but significantly elevated the expression (Figure 4C,D), suggesting that not only intramolecular copper but also the structure of EDTC is required for inducing endothelial *syndecan-4* mRNA expression.

### 2.3. Cu(edtc)_2_ Induces Endothelial Syndecan-4 Expression via p38 MAPK Activation

**Figure 5:**
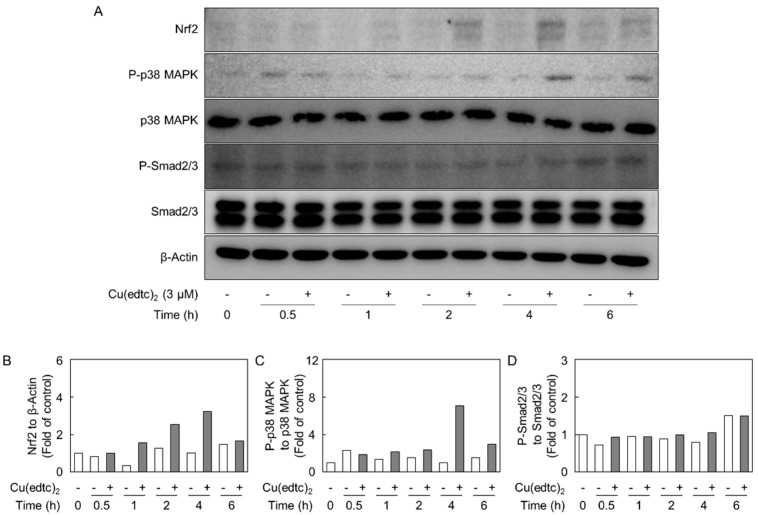
Effects of Cu(edtc)_2_ on the expression of NF-E2-related factor2 (Nrf2) and phosphorylation of p38 mitogen-activated protein kinase (MAPK) and Smad2/3 in vascular endothelial cells. The cells were incubated at 37 °C for 0.5, 1, 2, 4, and 6 h in the presence of Cu(edtc)_2_ (3 µM). (**A**) Cell lysates were then subjected to western blotting; (**B**–**D**) the ratio of the intensity of Nrf2, phosphorylated p38 MAPK (P-p38 MAPK), and phosphorylated Smad2/3 (P-Smad2/3) in (**A**) to those of β-Actin, p38 MAPK, and Smad2/3, respectively; values are means of two replicates from two independent experiments (**B**–**D**).

Because we recently reported that the Smad2/3-p38 MAPK pathway is involved in the upregulation of syndecan-4 by transforming growth factor-β_1_ (TGF-β_1_) in vascular endothelial cells [21], we analyzed the role of this pathway in Cu-10-mediated *syndecan-4* induction. Figure 5 shows the time course of the expression of Nrf2, p38 MAPK, and Smad2/3 in vascular endothelial cells after treatment with Cu(edtc)_2_. The copper complex increased the expression of Nrf2 (at approximately 110 kDa) after 1- to 6-h treatments as reported previously [18]. The phosphorylation of p38 MAPK (at approximately 38 kDa) also increased after a 1-h exposure to Cu(edtc)_2_. On the other hand, Cu(edtc)_2_ did not affect the phosphorylation of Smad2/3 (at approximately 52 and 48 kDa, respectively) at 6 h or less in vascular endothelial cells.

The activation of p38 MAPK by Cu(edtc)_2_ expression in Cu(edtc)_2_-treated vascular endothelial cells occurred after 1 h and longer (Figure 5), while an increase in the *syndecan-4* mRNA expression occurred after 4 h and longer (Figure 1C). These results suggest that activation of the downstream effectors of p38 MAPK is required for the induction of endothelial *syndecan-4*.

**Figure 6:**
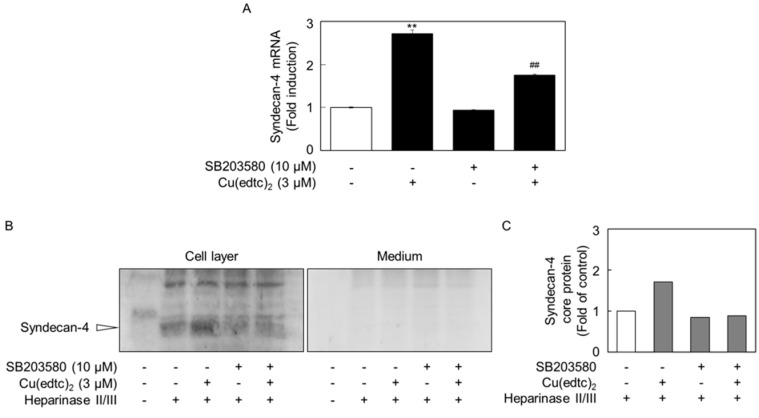
A p38 MAPK inhibitor SB203580 suppresses the syndecan-4 induction by Cu(edtc)_2_ in vascular endothelial cells. The expression of syndecan-4 (**A**) mRNA and (B) core protein; values are means ± S.E. of four replicates. ** *p* < 0.01 vs. control; ^##^ *p* < 0.01 vs. Cu(edtc)_2_. The position of syndecan-4 core protein is indicated by the arrowhead; (**C**) the intensity of the blots of cell layer in (**B**); the cells were treated with Cu(edtc)_2_ (3 µM) for 8 h after pretreatment with of SB203580 (10 µM) for 3 h. Values are means of two replicates from two independent experiments (**C**).

To examine the possibility that activation of p38 MAPK is involved in the upregulation of syndecan-4 expression by Cu(edtc)_2_, vascular endothelial cells were pretreated with the p38 MAPK inhibitor SB203580 and then treated with Cu(edtc)_2_ (Figure 6). The inhibitor partly suppressed Cu(edtc)_2_-induced *syndecan-4* mRNA expression (Figure 6A). In addition, Cu(edtc)_2_ increased expression of the syndecan-4 core protein (at approximately 35 kDa) in the cell layer and this increase was diminished when the cells were pretreated with the p38 MAPK inhibitor before treatment with Cu(edtc)_2_ (Figure 6B,C).

**Figure 7:**
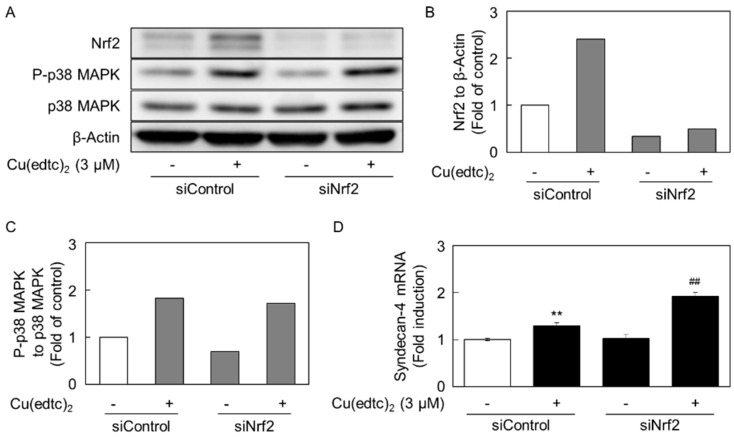
Nrf2 does not contribute to the activation of p38 MAPK and induction of *syndecan-4* by Cu(edtc)_2_ in vascular endothelial cells; (**A**) effect of Nrf2 on the activation of p38 MAPK by Cu(edtc)_2_; (**B**,**C**) the ratio of the intensity of Nrf2 and P-p38 MAPK in (**A**) to those of β-Actin and p38 MAPK, respectively; (**D**) expression of *syndecan-4* mRNA. siRNA-transfected cells were treated with Cu(edtc)_2_ (3 µM) for 8 h. Values are means of two replicates from two independent experiments (**B**,**C**); ** *p* < 0.01 vs. control; ^##^ *p* < 0.01.

The involvement of Nrf2 in the phosphorylation of p38 MAPK and induction of syndecan-4 expression was investigated (Figure 7) because Cu(edtc)_2_ is a potent inducer of Nrf2 expression [18]. However, the siRNA-mediated suppression of Nrf2 expression did not affect either the phosphorylation of p38 MAPK (Figure 7A–C) or the induction of *syndecan-4* expression by Cu(edtc)_2_ (Figure 7D). Moreover, the Cu(edtc)_2_-induced expression of *syndecan-4* was enhanced by the Nrf2 suppression. This may be caused by an induction of syndecan-4 expression via p-38 MAPK-independent pathways activated by some stress due to a lower expression of Nrf2.

**Figure 8:**
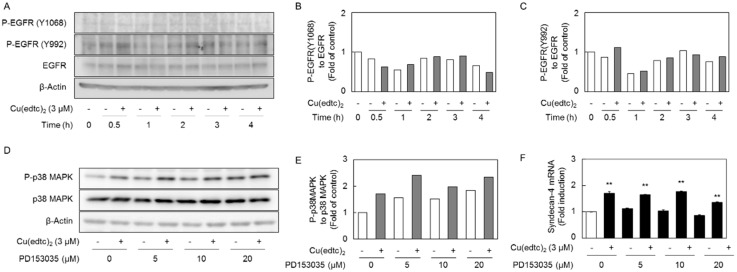
Epidermal growth factor receptor (EGFR) activation does not contribute to the Cu(edtc)_2_-mediated activation of p38 MAPK and induction of *syndecan-4* in vascular endothelial cells. (**A**) Activation of EGFR by Cu(edtc)_2_; the cells were incubated at 37 °C for 0.5, 1, 2, 3, and 4 h in the presence of Cu(edtc)_2_ (3 µM), then lysates were subjected to Western blotting. The ratio of the intensity of (**B**) phosphorylated EGFR (P-EGFR)(Y1068) and (**C**) P-EGFR(Y992) in (**A**) to that of EGFR; (**D**) EGFR-dependent p38 MAPK activation. The cells were treated with Cu(edtc)_2_ (3 µM) for 4 h after pretreatment with an EGFR inhibitor PD153035 (5, 10, and 20 µM) for 1 h. Lysates were then subjected to immunoblotting. (**E**) The ratio of the intensity of P-p38 MAPK to that of p38 MAPK in (**D**); (**F**) expression of *syndecan-4* mRNA. The cells were treated with Cu(edtc)_2_ (3 µM) for 8 h after pretreatment with of PD153035 (5, 10, and 20 µM) for 1 h, then *syndecan-4* mRNA levels were determined by RT-PCR. Values are means of two replicates from two independent experiments (**B**,**C**,**E**).

Since we recently reported that epidermal growth factor receptor (EGFR) autophosphorylation is a critical pathway for the activation of p38 MAPK in vascular endothelial cells treated with methylmercury [22], we next examined the involvement of EGFR activation in the Cu10-mediated activation of p38 MAPK in the cells (Figure 8) because both Cu(edtc)_2_ and methylmercury are electrophiles. It was revealed that Cu(edtc)_2_ enhanced EGFR autophosphorylation (at approximately 135 kDa) at the site of Y992 but not Y1068 at 0.5 h after treatment with Cu(edtc)_2_ (Figure 8A–C). However, pretreatment with the EGFR inhibitor PD153035 failed to suppress the Cu(edtc)_2_-mediated activation of p38 MAPK and induction of *syndecan-4* mRNA expression in the cells (Figure 8D–F).

## 3. Discussion

We believe that metal coordination complexes are a promising tool to analyze biological systems because of their unique three-dimensional structures [17]. In fact, we found that the inhibition of prolyl hydroxylase-domain-containing protein 2 induces endothelial syndecan-4 expression through the activation of the hypoxia-inducible factor-1α/β using 1,10-phenanthroline with or without zinc or rhodium [23]. Cu(edtc)_2_ is a metal coordination complex that is known to exhibit several biological activities in cultured vascular endothelial cells. Cu(edtc)_2_ inhibits the synthesis of tissue plasminogen activator [20] and reduces the fibrinolytic activity of the conditioned medium of the cells. The copper complex increases the expression of Nrf2 [18] and this activation is involved in the induction of *MT-1A*, a subisoform of metallothionein [19]. We therefore hypothesized that Cu(edtc)_2_ could modulate the synthesis of anticoagulant proteoglycans. It was found that Cu(edtc)_2_ induces the synthesis of syndecan-4, an anticoagulant transmembrane type of small heparan sulfate proteoglycan. The expression of *syndecan-4* mRNA alone was markedly increased by Cu(edtc)_2_; the expression of *syndecan-2* and *biglycan* was slightly increased, that of *perlecan* and *syndecan-3* was unchanged, and that of *syndecan-1* was decreased, indicating that Cu(edtc)_2_ is a good tool to analyze the mechanisms underlying the upregulation of syndecan-4 in vascular endothelial cells. At the same time, this study revealed that Cu(edtc)_2_ can modulate endothelial proteoglycan synthesis as well as fibrinolytic protein synthesis [20] and metallothionein induction [19].

The present results suggested that Cu(edtc)_2_ specifically induces the expression of syndecan-4 via the activation of p38 MAPK, independent of Nrf2 and EGFR, in vascular endothelial cells and that the whole structure of Cu(edtc)_2_ is required for this induction. Recently, we found that TGF-β_1_ upregulates the expression level of syndecan-4 in vascular endothelial cells by activating the Smad2/3-p38 MAPK pathway [21,24]. Since Cu(edtc)_2_ did not activate Smad2/3, it is postulated that the activation of p38 MAPK, which mediates the induction of syndecan-4 by Cu(edtc)_2_, does not require the activation of Smad2/3. In other words, the induction of endothelial syndecan-4 expression by Cu(edtc)_2_ is not mediated by the upregulation of TGF-β. Clarification of the mechanisms underlying the activation of p38 MAPK by Cu(edtc)_2_ is a future problem.

We recently found that Cu(edtc)_2_ inhibits the proteasome but increases the expression of Nrf2 in vascular endothelial cells [18]. In this report, it was shown that copper complexes **1**–**3** accumulate more than Cu(edtc)_2_ in cells but the expression of Nrf2 increased only after treatment with either Cu(edtc)_2_ or copper complex **1**, to a similar degree. In this study, it was shown that Cu(edtc)_2_, but not copper complex **1**, strongly induces the transcriptional induction of endothelial *syndecan-4*, suggesting that Nrf2 is not involved in this induction. On the other hand, the data suggested that the Cu(edtc)_2_-induced expression of endothelial syndecan-4 is mediated by the activation of p38 MAPK. Although the mechanisms underlying the activation of p38 MAPK by Cu(edtc)_2_ are unclear, an assumption can be made that the inhibition of the proteasome is involved in this activation because several proteasome inhibitors such as bortezomib [25], MG132 [26], lactacystin [27], epoxomicin [28], benzyloxycarbonyl-leucyl-leucyl-phenylalaninal [29], and acetyl-leucyl-leucyl-norleucinal [30] promote the phosphorylation of p38 MAPK, and Cu(edtc)_2_ is a protease inhibitor [18]. On the other hand, it has been reported that the activation of nuclear factor-κB (NF-κB) induces syndecan-4 expression in endothelial cells [31] and nucleus pulposus cells [32]. However, since proteasome inhibitors prevent the activation and translocation of NF-κB [33,34], it is unlikely that the activation of p38 MAPK by Cu(edtc)_2_, a signal to induce endothelial syndecan-4, is mediated by NF-κB.

The present study revealed that a copper complex Cu(edtc)_2_ induces the expression of syndecan-4, a small heparan sulfate proteoglycan, in vascular endothelial cells. This induction was mediated by the activation of p38 MAPK and required the whole structure of Cu(edtc)_2_. Although the mechanisms underlying Cu(edtc)_2_-mediated p38 MAPK activation remain to be elucidated, the present data showed for the first time that p38 MAPK is importantly involved in regulating endothelial syndecan-4 expression. Additionally, the present study and our previous studies [18,19,20,21,23] support the hypothesis that metal coordination complexes including Cu(edtc)_2_ are good tools for analyzing biological systems. Bioorganometallics is a research field in which metal coordination complexes are used as such tools [17,35]. Further studies based on this strategy will be required to investigate unknown biological systems.

## 4. Materials and Methods

### 4.1. Materials

Bovine aortic endothelial cells were purchased from Cell Applications (San Diego, CA, USA). Dulbecco’s modified Eagle’s medium (DMEM) and Ca^2+^- and Mg^2+^-free phosphate-buffered saline were obtained from Nissui Pharmaceutical (Tokyo, Japan). Fetal bovine serum (FBS) was purchased from Biosera (Kansas, MO, USA). Tissue culture dishes and plates were from AGC Techno Glass (Shizuoka, Japan). Copper(II) diacetate was purchased from Sigma-Aldrich. Ammonium 1-pyrrolidinecarbodithioate, Cu(edtc)_2_, Zn(edtc)_2_, Ni(edtc)_2_, Fe(edtc)_3_, and zinc(II) bis(*N*-ethyl-*N*-phenyldithiocarbamate) were purchased from Tokyo Chemical Industry (Tokyo, Japan). Copper complex **1**, CuSO_4_, Na(edtc), and Immunostar Basic were obtained from Wako Pure Chemical Industries (Osaka, Japan). A p38 MAPK inhibitor SB203580 and EGFR inhibitor PD153035 were purchased from Cayman Chemical (Ann Arbor, MI, USA) and Calbiochem (Boston, MA, USA), respectively. QIAzol lysis reagent was purchased from QIAGEN (Valencia, CA, USA). GeneAce SYBR qPCR mix α was obtained from Nippon Gene (Tokyo, Japan). Lipofectamine RNAiMAX, high-capacity cDNA reverse transcription kit and BCA protein assay kit were purchased from Thermo Fisher Scientific (Waltham, MA, USA). Anti-phospho-p38 MAPK (#9211), anti-p38 MAPK (#9212), anti-phospho-Smad2/3 (#8828), anti-Smad2/3 (#8685), anti-phospho-EGFR Y992 (#2235), horseradish peroxidase-conjugated anti-rabbit IgG (#7074), and anti-mouse IgG (#7076) were obtained from Cell Signaling Technology (Beverly, MA, USA). Anti-EGFR antibody (MI-12-1) was purchased from Medical & Biological Laboratories (Aichi, Japan). Anti-syndecan-4 (sc-9497) and anti-Nrf2 (sc-13032) antibodies were purchased from Santa Cruz Biotechnology (Santa Cruz, CA, USA). Anti-phospho-EGFR Y1068 (ab32430) and horseradish peroxidase-conjugated anti-goat IgG antibody (ab6885) were obtained from Abcam (Bristol, UK). Heparinase II (derived from *Flavobacterium heparinum*) and heparinase III (EC 4.2.2.8, derived from *F. heparinum*) were purchased from IBEX Technologies (Montreal, QC, Canada). Diethylaminoethyl–Sephacel and PVDF Immobilon-P membranes (pore size 0.45 µm) were purchased from Merck KGaA (Darmstadt, Germany). Other reagents of the highest grade available were obtained from Nacalai Tesque (Kyoto, Japan).

### 4.2. Synthesis

Copper complexes **2** and **3** were synthesized as previously described [18]. Copper complex **4** was analogously synthesized as follows: in a 500-mL round bottomed flask, a biphasic mixture of copper(II) diacetate (3.63 g, 20 mmol), zinc(II) bis(*N*-ethyl-*N*-phenyldithiocarbamate) (9.16 g, 20 mmol), CH_2_Cl_2_ (150 mL), water (150 mL), and 25% aqueous ammonia (50 mL) was stirred at room temperature (rt) for 1 h under air. The organic layer was separated and the aqueous layer was extracted with CH_2_Cl_2_ (*ca.* 20 mL). The combined, black organic layer was washed with water, concentrated under reduced pressure, concentrated again after adding toluene (5 mL), and dried under vacuum at 60 °C to yield the desired product as black solids (9.09 g, >99%). Elemental analysis calcd for [C_18_H_20_CuN_2_S_4_]: C, 47.39; H, 4.42; N, 6.14; found: C, 47.98; H, 4.38; N, 6.13. Copper complex **5** was prepared as follows: in a 500-mL round bottomed flask, a biphasic mixture of copper(II) diacetate (3.63 g, 20 mmol), ammonium 1-pyrrolidinecarbodithioate (6.57 g, 40 mmol), and water (300 mL) was stirred at rt overnight under air. The resulting dark-brown suspension was extracted with CH_2_Cl_2_ (*ca.* 500 mL). The combined, black organic layer was washed with water (200 mL), concentrated under reduced pressure, concentrated again after adding toluene (5 mL), and dried under vacuum at 60 °C to yield the desired product as dark-gray solids (6.36 g, 89%). Elemental analysis calcd for [C_18_H_20_CuN_2_S_4_]: C, 33.73; H, 4.54; N, 7.87; found: C, 34.66; H, 4.54; N, 7.87. These complexes are known compounds [36]. The elemental analyses were recorded on a Yanaco CHN recorder MT-6 at the Chemical Instrumental Center, Research Center for Materials Science, Nagoya University.

### 4.3. Cell Culture and Treatments

Vascular endothelial cells were cultured in a humidified atmosphere of 5% CO_2_ at 37 °C in DMEM supplemented with 10% FBS until confluent. They were then transferred to 35- or 100-mm dishes and cultured until confluent. The medium was then discarded and the cells were washed twice with fresh serum-free DMEM; the cells were treated with Cu(edtc)_2_, CuSO_4_, Na(edtc), or other organometallic compounds (0.5, 1, 2, 3, 5, or 6 µM) for 0.5, 1, 2, 4, 6, 8, 12, or 24 h in the presence or absence of p38 MAPK inhibitor SB203580 (10 µM) or EGFR inhibitor SP153035 (5, 10, or 20 µM) in fresh serum-free DMEM.

### 4.4. siRNA Transfection

The transfection of siRNAs was performed using Lipofectamine RNAiMAX according to the manufacturer’s protocol. Briefly, annealed siRNA duplexes and Lipofectamine RNAiMAX were dissolved in Opti-MEM in separate tubes and incubated for 5 min at rt, followed by mixing and incubation for 20 min at rt. Vascular endothelial cells were grown to near subconfluence in DMEM supplemented with 10% FBS and then incubated at 37 °C in fresh DMEM supplemented with 10% FBS in the presence of the siRNA/Lipofectamine RNAiMAX mixture. The final concentrations of siRNA and Lipofectamine RNAiMAX were 18 nM and 0.09%, respectively. After 4 h, the medium was changed to DMEM supplemented with 10% FBS and the cells were incubated at 37 °C for 18 h. The medium was then changed to DMEM, and the cells were treated for 8 h with Cu(edtc)_2_ (3 µM). The siRNA sequences were as follows: bovine Nrf2 siRNA (siNrf2), 5′-CCAUUGAUCUCUCUGAUCUdTdT-3′ (sense), and 5′-AGAUCAGAGAGAUCAAUGGGC-3′ (antisense). Negative control siRNA (siControl; Qiagen, Valencia, CA, USA) was used as a nonspecific sequence.

### 4.5. Real-Time RT-PCR

Total RNA was extracted from vascular endothelial cells as described previously [23]. Complementary DNA was synthesized from the mRNA using a high-capacity cDNA reverse transcription kit. RT-PCR was performed in 20 µL per well using GeneAce SYBR qPCR mix α with 1 ng cDNA and 0.1 µM primers (Table 1) in a StepOnePlus real-time PCR system (Thermo Fisher Scientific). Levels of mRNAs encoding *perlecan*, *syndecan-1*, *syndecan-2*, *syndecan-3*, *syndecan-4*, *biglycan*, *decorin*, and *glyceraldehyde-3-phosphate dehydrogenase* (*GAPDH*) were quantified using the relative standard curve method.

### 4.6. Proteoglycan Core Protein Extraction and Western Blot Analysis

Proteoglycans were extracted from the cell layer and the conditioned medium of vascular endothelial cells under dissociative conditions and concentrated as previously described [23]. Concentrated proteoglycans were dissolved with 0.02 IU/mL heparinase II/III in 100 mM Tris-HCl buffer (pH 7.0) containing 10 mM calcium acetate and 18 mM sodium acetate for 3 h at 37 °C to determine the core proteins of syndecan-4. The proteoglycans were lysed in sodium dodecyl sulfate (SDS) sample buffer ((50 mM Tris-HCl buffer solution containing 2% SDS and 10% glycerol (pH 6.8)), followed by incubation at 95 °C for 3 min. Proteoglycans were separated by SDS-polyacrylamide gel electrophoresis on a 10% polyacrylamide gel and transferred onto an Immobilon-P membrane at 2 mA/cm^2^ for 1 h. Membranes were blocked for 1 h with 5% skim milk in 20 mM Tris-HCl buffer solution (pH 7.5) containing 150 mM NaCl and 0.1% Tween 20 (TTBS) or 2% BSA-TTBS solution, and incubated overnight with a primary antibody at 4 °C. The membranes were washed and incubated with horseradish peroxidase-conjugated secondary antibodies for 1 h at rt. Immunoreactive bands were visualized using the Immunostar Basic enhanced chemiluminescence Western blot detection reagent and scanned using a LAS 3000 Imager (Fujifilm, Tokyo, Japan). Representative blots are shown from two independent experiments.

### 4.7. Statistical Analysis

Data were analyzed for statistical significance by Student’s *t*-test, Dunnet’s or Tukey’s method, when possible. *p* values less than 0.01 were considered statistically significant.

## 5. Conclusions

In the present study, it was revealed that copper(II) bis(diethyldithiocarbamate), termed Cu(edtc)_2_, induces the expression of syndecan-4, a transmembrane heparan sulfate proteoglycan, in vascular endothelial cells. This activity of Cu(edtc)_2_ requires both the diethyldithiocarbamate structure as the ligand and an intramolecular copper atom. Cu(edtc)_2_-mediated syndecan-4 induction depends on the activation of p38 MAPK; however, the Smad2/3, Nrf2, and EGFR pathways were not involved in the induction. The present study succeeded in clarifying, for the first time, an important role of p38 MAPK in the synthesis of syndecan-4 in vascular endothelial cells, using metal coordination complexes.

## Figures and Tables

**Table 1:** Bovine gene-specific primers for RT-PCR.

Gene	Forward Primer (5′-3′)	Reverse Primer (5′-3′)
*Perlecan*	ATGGCAGCGATGAAGCGGAC	TTGTGGACACGCAGCGGAAC
*Syndecan-1*	CAGTCAGGAGACAGCATCAG	CCGACAGACATTCCATACC
*Syndecan-2*	CCAGATGAAGAGGACACAAACG	CCAATAACTCCGCCAGCAA
*Syndecan-3*	CAAGCAGGCGAGCGTC	GGTGGCAGAGATGAAGTGG
*Syndecan-4*	TTGCCGTCTTCCTCGTGC	AGGCGTAGAACTCATTGGTGG
*Biglycan*	GCTGCCACTGCCATCTGAG	CGAGGACCAAGGCGTAG
*GAPDH*	AACACCCTCAAGATTGTCAGCAA	ACAGTCTTCTGGGTGGCAGTGA

## Abbreviations

Cu(edtc)_2_	Copper(II) bis(diethyldithiocarbamate)
Fe(edtc)_3_	Iron(III) tris(diethyldithiocarbamate)
Ni(edtc)_2_	Nickel(II) bis(diethyldithiocarbamate)
Na(edtc)	Sodium diethyldithiocarbamate
Zn(edtc)_2_	Zinc(II) bis(diethyldithiocarbamate)

## References

[1] Ruoslahti E. (1988). Structure and biology of proteoglycans. Annu. Rev. Cell Biol..

[2] Iozzo R.V., Schaefer L. (2015). Proteoglycan form and function: A comprehensive nomenclature of proteoglycans. Matrix Biol..

[3] Mitsou I., Multhaupt H.A.B., Couchman J.R. (2017). Proteoglycans, ion channels and cell-matrix adhesion. Biochem. J..

[4] De Agostini A.I., Watkins S.C., Slayter H.S., Youssoufian H., Rosenberg R.D. (1990). Localization of anticoagulantly active heparan sulfate proteoglycans in vascular endothelium: Antithrombin binding on cultured endothelial cells and perfused rat aorta. J. Cell Biol..

[5] Kojima T. (2002). Targeted gene disruption of natural anticoagulant proteins in mice. Int. J. Hematol..

[6] Lin M.C., Lin C.F., Li C.F., Sun D.P., Wang L.Y., Hsing C.H. (2015). Anesthetic propofol overdose causes vascular hyperpermeability by reducing endothelial glycocalyx and ATP production. Int. J. Mol. Sci..

[7] Camejo G. (1982). The interaction of lipids and lipoproteins with the intercellular matrix of arterial tissue: Its possible role in atherogenesis. Adv. Lipid Res..

[8] Paulsson M. (1992). Basement membrane proteins: Structure, assembly, and cellular interactions. Crit. Rev. Biochem. Mol. Biol..

[9] Yamamoto C., Deng X., Fujiwara Y., Kaji T. (2005). Proteoglycans predominantly synthesized by human brain microvascular endothelial cells in culture are perlecan and biglycan. J. Health Sci..

[10] Saku T., Furthmayr H. (1989). Characterization of the major heparan sulfate proteoglycan secreted by bovine aortic endothelial cells in culture. Homology to the large molecular weight molecule of basement membranes. J. Biol. Chem..

[11] Kojima T., Shworak N.W., Rosenberg R.D. (1992). Molecular cloning and expression of two distinct cDNA-encoding heparan sulfate proteoglycan core proteins from a rat endothelial cell line. J. Biol. Chem..

[12] Järveläinen H.T., Kinsella M.G., Wight T.N., Sandell L.J. (1991). Differential expression of small chondroitin/dermatan sulfate proteoglycans, PG-I/biglycan and PG-II/decorin, by vascular smooth muscle and endothelial cells in culture. J. Biol. Chem..

[13] Shworak N.W., Kojima T., Rosenberg R.D. (1993). Isolation and characterization of ryudocan and syndecan heparan sulfate proteoglycans, core proteins, and cDNAs from a rat endothelial cell line. Haemostasis.

[14] Baeyens N., Mulligan-Kehoe M.J., Corti F., Simon D.D., Ross T.D., Rhodes J.M., Wang T.Z., Mejean C.O., Simons M., Humphrey J. (2014). Syndecan 4 is required for endothelial alignment in flow and atheroprotective signaling. Proc. Natl. Acad. Sci. USA.

[15] Ishiguro K., Kadomatsu K., Kojima T., Muramatsu H., Iwase M., Yoshikai Y., Yanada M., Yamamoto K., Matsushita T., Nishimura M. (2001). Syndecan-4 deficiency leads to high mortality of lipopolysaccharide-injected mice. J. Biol. Chem..

[16] Matsui Y., Ikesue M., Danzaki K., Morimoto J., Sato M., Tanaka S., Kojima T., Tsutsui H., Uede T. (2011). Syndecan-4 prevents cardiac rupture and dysfunction after myocardial infarction. Circ. Res..

[17] Fujie T., Hara T., Kaji T. (2016). Toxicology of organic-inorganic hybrid molecules: Bio-organometallics and its toxicology. J. Toxicol. Sci..

[18] Fujie T., Murakami M., Yoshida E., Tachinami T., Shinkai Y., Fujiwara Y., Yamamoto C., Kumagai Y., Naka H., Kaji T. (2016). Copper diethyldithiocarbamate as an activator of Nrf2 in cultured vascular endothelial cells. J. Biol. Inorg. Chem..

[19] Fujie T., Segawa Y., Yoshida E., Kimura T., Fujiwara Y., Yamamoto C., Satoh M., Naka H., Kaji T. (2016). Induction of metallothionein isoforms by copper diethyldithiocarbamate in cultured vascular endothelial cells. J. Toxicol. Sci..

[20] Fujie T., Okino S., Yoshida E., Yamamoto C., Naka H., Kaji T. (2017). Copper diethyldithiocarbamate as an inhibitor of tissue plasminogen activator synthesis in cultured human coronary endothelial cells. J. Toxicol. Sci..

[21] Hara T., Yoshida E., Fujiwara Y., Yamamoto C., Kaji T. (2017). Transforming growth factor-beta1 modulates the expression of syndecan-4 in cultured vascular endothelial cells in a biphasic manner. J. Cell. Biochem..

[22] Yoshida E., Kurita M., Eto K., Kumagai Y., Kaji T. (2017). Methylmercury promotes prostacyclin release from cultured human brain microvascular endothelial cells via induction of cyclooxygenase-2 through activation of the EGFR-p38 MAPK pathway by inhibiting protein tyrosine phosphatase 1B activity. Toxicology.

[23] Hara T., Kojima T., Matsuzaki H., Nakamura T., Yoshida E., Fujiwara Y., Yamamoto C., Saito S., Kaji T. (2017). Induction of syndecan-4 by organic-inorganic hybrid molecules with a 1,10-phenanthroline structure in cultured vascular endothelial cells. Int. J. Mol. Sci..

[24] Hara T., Yoshida E., Shinkai Y., Yamamoto C., Fujiwara Y., Kumagai Y., Kaji T. (2017). Biglycan intensifies ALK5-Smad2/3 signaling by TGF-beta1 and downregulates syndecan-4 in cultured vascular endothelial cells. J. Cell. Biochem..

[25] Yu C., Rahmani M., Conrad D., Subler M., Dent P., Grant S. (2003). The proteasome inhibitor bortezomib interacts synergistically with histone deacetylase inhibitors to induce apoptosis in Bcr/Abl+ cells sensitive and resistant to STI571. Blood.

[26] Shibata T., Imaizumi T., Matsumiya T., Tamo W., Hatakeyama M., Yoshida H., Munakata H., Fukuda I., Satoh K. (2003). Effect of MG132, a proteasome inhibitor, on the expression of growth related oncogene protein-alpha in human umbilical vein endothelial cells. Cytokine.

[27] Yamamoto N., Sawada H., Izumi Y., Kume T., Katsuki H., Shimohama S., Akaike A. (2007). Proteasome inhibition induces glutathione synthesis and protects cells from oxidative stress: Relevance to parkinson disease. J. Biol. Chem..

[28] Fernandes A.F., Bian Q., Jiang J.K., Thomas C.J., Taylor A., Pereira P., Shang F. (2009). Proteasome inactivation promotes p38 mitogen-activated protein kinase-dependent phosphatidylinositol 3-kinase activation and increases interleukin-8 production in retinal pigment epithelial cells. Mol. Biol. Cell.

[29] Shi Y.Y., Small G.W., Orlowski R.Z. (2006). Proteasome inhibitors induce a p38 mitogen-activated protein kinase (MAPK)-dependent anti-apoptotic program involving MAPK phosphatase-1 and Akt in models of breast cancer. Breast Cancer Res. Treat..

[30] Honma Y., Shimizu S., Takehara T., Harada M. (2014). Sorafenib enhances proteasome inhibitor-induced cell death via inactivation of Akt and stress-activated protein kinases. J. Gastroenterol..

[31] Okuyama E., Suzuki A., Murata M., Ando Y., Kato I., Takagi Y., Takagi A., Murate T., Saito H., Kojima T. (2013). Molecular mechanisms of syndecan-4 upregulation by TNF-alpha in the endothelium-like EAhy926 cells. J. Biochem..

[32] Yang H., Liu H., Li X., Pan H., Li Z., Wang J., Zheng Z. (2015). TNF-alpha and TGF-beta1 regulate syndecan-4 expression in nucleus pulposus cells: Role of the mitogen-activated protein kinase and NF-kappaB pathways. Connect. Tissue Res..

[33] Lovborg H., Oberg F., Rickardson L., Gullbo J., Nygren P., Larsson R. (2006). Inhibition of proteasome activity, nuclear factor-kappaB translocation and cell survival by the antialcoholism drug disulfiram. Int. J. Cancer.

[34] Traenckner E.B., Wilk S., Baeuerle P.A. (1994). A proteasome inhibitor prevents activation of NF-kappa B and stabilizes a newly phosphorylated form of I kappa B-alpha that is still bound to NF-kappa B. EMBO J..

[35] Jaouen G. (2006). Bioorganometallics.

[36] Hendrickson A.R., Martin R.L., Rohde N.M. (1976). Dithiocarbamates of copper (I), copper (II), and copper (III). An electrochemical study. Inorg. Chem..

